# Hybrid Composites of Polylactide with Basalt and Carbon Fibers and Their Thermal Treatment

**DOI:** 10.3390/ma12010095

**Published:** 2018-12-28

**Authors:** Stanisław Kuciel, Paulina Romańska

**Affiliations:** Faculty of Mechanical Engineering, Cracow University of Technology, ul. Warszawska 24, 31-155 Cracow, Poland; stask@mech.pk.edu.pl

**Keywords:** biopolymer, bio-based, structural biocomposites, natural fiber, cold crystallization

## Abstract

In this research, polylactide was hybrid-reinforced and heat-treated in order to obtain durable structural materials with the use of eco-friendly components. Differential scanning calorimetry (DSC) analysis, tensile tests at various temperatures, Vicat tests, impact tests, and microscopic observations were conducted on the annealed and non-heat-treated specimens. The theoretical and true density, as well as water absorption, were also determined. The simultaneous introduction of chopped carbon and basalt fibers in equal mass fractions of 7.5% and 12.5% resulted in satisfactory increases in stiffness and tensile strength. The reinforcing effect was more efficient for the heat-treated composites, especially at elevated temperatures. Heat treatment significantly increased the degree of crystallinity of the matrix, improving heat resistance and reducing water absorption. It also reduced the stress concentrations in the injection-molded specimens.

## 1. Introduction

Numerous publications have been devoted to polymer materials based on polylactide (PLA). However, in those publications, attention was mainly focused on the biomedical applications of this material or on the aspect of its biodegradability. Less often was it considered in terms of the possible structural applications. This study focuses on the assessment of the applicability of PLA as a matrix of hybrid composites intended for the production of rigid and durable technical injection-molded parts. At this point, it should be added that so far, reports on hybrid composites with carbon and basalt fibers have appeared in only a few publications, and concerned laminates with a thermoset matrix [1,2,3].

While carbon fiber has a well-established position as a reinforcing filler in both thermoset and thermoplastic polymer composites, basalt fiber is a relatively new filler for thermoplastics. Basalt fiber is obtained in a process similar to the glass fiber production, but its manufacture does not require the incorporation of components that are harmful to health and the environment, such as boron and fluorine oxides, which are present in the majority of E-type glass fibers [4,5]. Here, only naturally occurring basalt rocks are the charge. For this reason, basalt fiber is sometimes classified as natural fiber, although it is certainly a great simplification. It should be considered as a material obtained entirely from natural, though not renewable, raw materials. In terms of basic oxide composition, the chemical composition of basalt fiber is similar to the composition of synthetic glass fiber. However, the main difference is a significant fraction of iron in basalt. In the oxide composition, the following oxides are predominant: SiO_2_, Al_2_O_3_, CaO, MgO, Fe_2_O, and FeO [6]. The strength properties of high-quality basalt fiber are intermediate between E-type and S-type glass fibers [7]. The other beneficial properties of basalt fiber include a high vibration-damping capacity, thermal and acoustic insulation, as well as high hardness and abrasion resistance [6,7,8,9,10,11,12]. Basalt fiber also has good resistance to the effect of atmospheric agents, alkalis, and acids. Compared to glass fiber, it is characterized by high resistance to the effect of strong alkalis, but rather modest resistance to the effect of strong acids [8]. The density of basalt fiber (2.6–2.7 g/cm^3^) is close to the density of glass fiber (2.5–2.6 g/cm^3^) [6]. As an environmentally friendly material with advantageous mechanical properties, basalt fiber seems to be a very good reinforcing filler for composites based on a bio-based polylactide. It is particularly preferred as a replacement for glass fiber.

Basalt fiber was initially obtained and optimized as a component for concrete reinforcement [13,14], and up-to-date construction still remains the main sector of its application. It is used, among other applications, in the form of a continuous filament for the production of composite rods and in the form of chopped fiber for the direct reinforcement of concrete. Many studies have also been devoted to polymer laminates reinforced with continuous basalt fiber. Starting from the 1980s, research has been carried out on the use of basalt fabrics in the production of composites based on polyester, epoxy, vinyl ester, or phenol resins [12,15,16,17,18,19,20]. The factor that is emphasized in most of these works is the competitiveness or predominance of basalt fiber over glass fiber as a component that allows obtaining better strength parameters under both static and dynamic loads.

Compared to the available literature on basalt fiber as a material reinforcing thermoset resins and concretes, there are only several publications that have discussed the subject of thermoplastic composites reinforced with this fiber [21,22,23,24]. Few publications are also available on polylactide-basalt composites [25,26,27,28,29]. In spite of this, even the results of the quoted works show the large potential of basalt fiber as reinforcing filler, especially for a semi-crystalline matrix.

In this research work, the authors’ attention was focused on polylactic hybrid composites with short fibers. When assessing the possibility of using new composites as technical materials, the functional properties of polylactide with basalt and carbon fibers were compared for the condition before and after heat treatment, affecting the degree of crystallinity in the materials tested. The total content of glass and basalt fibers in the new composites was 15 wt % and 25 wt %.

## 2. Materials and Methods 

### 2.1. Materials

PLA properties depend on the stereochemical composition of the repeating units and their distribution along the chain. The polylactide that was selected as a matrix of new technical hybrid composites is a material with the trade name Ingeo 2500HP, which was produced by Nature Works. It is a PLA with very low D-isomer content, which is capable of crystallization. It was reinforced by introducing glass and basalt fibers in the same percent by weight, i.e. the total fiber content was 15 wt % and 25 wt %. The chopped carbon fibers that were used in this study were PX35 type 45 fibers produced by Zoltek Corporation (Nyergesujfalu, Hungary) with a nominal diameter of 7.2 μm and a nominal cut length of six mm (according to the manufacturer’s declaration). These fibers should be classified as HT-type (High Tensile) carbon fibers, which are characterized by a standard modulus of elasticity and high tensile strength. The BCS17-6.4-KV16 chopped basalt fibers were supplied by Basaltex (Wevelgem, Belgium). The properties of these fibers are given in Table 1.

Composite granulates whose composition is given in Table 2 were manufactured using a line for compounding equipped with a MARIS TM30 co-rotating twin screw extruder (Maris America Corp., Windsor Mill, MD, USA). Standard dumbbell-shaped specimens were produced by injection molding on an Engel ES 200/40 HSL injection molding machine (ENGEL GmbH, Schwertberg, Austria). The processes of compounding and injection were carried out in a Laboratory of Plastics Technology operating under Grupa Azoty SA in Tarnów, Poland. The injection parameters are described in Table 3. The same injection temperature profile was applied for all of the materials. 

The examined materials were injected into a cold mold to prevent their crystallization and, at the same time, significantly shorten the time of the cycle. Some of the specimens obtained in this way were heat treated, holding them in a furnace at 100 °C for 45 min. During annealing, specimens were placed between two glass plates to prevent their deformation.

### 2.2. Methods of Testing

#### 2.2.1. DSC Analysis

Differential scanning calorimetry (DSC) tests were performed using a Mettler Toledo DSC1 analyzer (METTLER TOLEDO, Columbus, OH, USA). The measurements were made on the specimens of 8.4–8.7 mg obtained from a central part of the injection-molded standard dumbbell-shape specimens in the temperature range between 25–200 °C under nitrogen atmosphere (30 mL/min). All of the measurements were taken at a heating and cooling rate of 10 °C/min, according to the following program: heating between 25–200 °C, annealing at 200 °C for 2 min, cooling between 200–25 °C, holding at 25 °C for 2 min, and second heating between 25–200 °C. An empty pan was used as a reference. 

Characteristic temperatures such as: glass transition temperature (*T_g_*), crystallization temperature (*T_c_*), cold crystallization temperature (*T_cc_*), and melting temperature (*T_m_*) were determined. The degree of crystallinity of neat PLA and the matrix in the composites was calculated using the following equation:

(1) $$\chi_{c}=\frac{\Delta H_{m}-\Delta H_{cc}-\Delta H_{rc}}{\Delta H_{m}^{0}\left(1-WF_{f}\right)}\cdot 100\%$$

where:

$\Delta H_{m}$—melting enthalpy (J/g)
$\Delta H_{cc}$—cold crystallization enthalpy (J/g)
$\Delta H_{rc}$—recrystallization enthalpy (J/g)
$\Delta H_{m}^{0}$—mean melting enthalpy of fully crystalline PLA, which equals 93.6 J/g [30]
$WF_{f}$—weight fraction of basalt and carbon fibers (15% or 25%)

#### 2.2.2. Determination of True and Theoretical Density

The density of PLA and PLA-based composites was determined using a RADWAG WAS 220/X electronic analytical balance (RADWAG Wagi Elektroniczne, Radom, Poland) with instrumentation. The density was determined by a hydrostatic weighing method. As a reference liquid with a known density, analytical grade ethanol was used. The density of the glass and basalt fibers that were used in this study was made available by fiber manufacturers. The respective values are given in Table 1. 

Knowing the assumed theoretical weight fraction of the filler and matrix in the tested composites, as well as the density of the fiber and matrix, the theoretical volume fraction of the fillers in the tested composites was calculated. For the tested hybrid composites with equal weight fractions of the basalt and carbon fibers, the volume fraction of basalt fiber was calculated using the following formula:

(2) $$VF_{f1}=\frac{1}{1+\frac{\rho_{f1}}{\rho_{f2}}+\frac{\rho_{f1}}{\rho_{m}}\left(\frac{1}{WF_{f1}}-2\right)}$$

where:

$\rho_{f1}$—density of basalt fiber
$\rho_{f2}$—density of carbon fiber
$\rho_{m}$—density of matrix
$WF_{f1}=WF_{f2}$—weight fraction of basalt fiber equal to carbon fiber wt. fraction (7.5% or 12.5%)

Volume fraction of carbon fiber ($VF_{f2}$) was calculated analogically.

Understanding the approximate theoretical volume fraction of components enabled the calculation of the theoretical density of the tested composites according to the basic law of mixtures:

(3) $$\rho_{c}=\rho_{f1}VF_{f1}+\rho_{f2}VF_{f2}+\rho_{m}\left(1-VF_{f1}-VF_{f2}\right)$$

In turn, a comparison of the theoretical density and true density allowed evaluating the correctness of component dosing and feeding in the process of producing composite granulates by compounding.

#### 2.2.3. Static Tensile Test

The static tensile test was carried out in accordance with ISO 527-1 on flat dumbbell-shaped specimens in the dry state with a traverse speed of five mm/min. Tests were carried out at −20 °C, 23 °C, and 55 °C on materials before and after heat treatment.

An MTS Criterion Model 43 testing machine (MTS Systems Corp., Eden Prairie, MN, USA) with maximum load range of up to 30 kN was used in the tests. For the accurate measurement of displacement allowing the determination of the elastic modulus, an MTS 634.31F axial extensometer was used. Machine control was carried out from the level of an MTS TestSuite TW software 1.0 (MTS Systems Corp., Eden Prairie, MN, USA), enabling the execution of precise and repeatable mechanical tests. The following properties were determined: tensile strength, tensile modulus of elasticity, and strain at break.

#### 2.2.4. Vicat Softening Temperature Measurement

Vicat softening temperature was measured at a heating rate of 50 °C/h using a Ceast HDT and Vicat Tester Type 6520 (CEAST, Pianezza, Italy) according to ISO 75. A load of 10 N was applied. 

#### 2.2.5. Charpy Impact Test

The tests were carried out in accordance with ISO 179 (method 1eU), on standardized specimens cut from the dumbbell-shaped specimens. The experiments were performed using Zwick/Roell HIT5.5P (Zwick Roell Group, Ulm, Germany) with a measuring range of up to 5 J.

#### 2.2.6. Fractographic Studies

A JEOL JSM5510LV scanning electron microscope (SEM, JEOL Ltd., Tokyo, Japan) was used in the tests. The specimen structure was examined on tensile fractures, and microscopic images were taken. All of the examinations were made on gold-sputtered fractures.

## 3. Results and Discussion

### 3.1. DSC Analysis 

**Figure 1:**
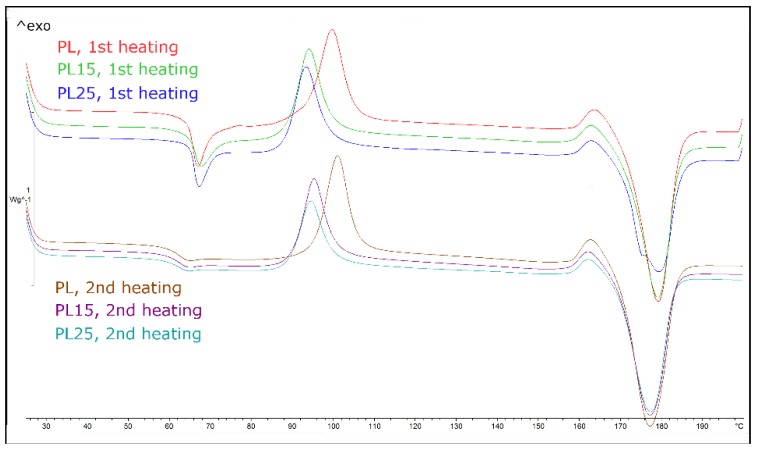
Differential scanning calorimetry (DSC) heating curves for non-heat-treated polylactide and its composites (first and second heating–cooling cycle).

**Figure 2:**
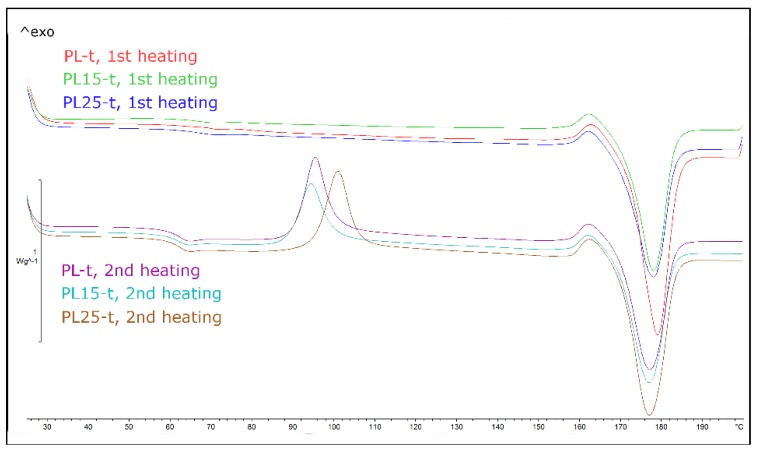
DSC heating curves for heat treated polylactide and its composites (first and second heating–cooling cycle).

The DSC tests allowed determining the structure of the sample material before and after heat treatment and confirmed the proper selection of the temperature at which the specimens were annealed during this treatment. Figure 1 illustrates the heating cycle (first and second pass) of PLA and the corresponding composites that had been non-heat-treated previously. Figure 2 shows the same curves for heat-treated materials. 

As it can be seen in Table 4, in heat-treated PLA specimens, cold crystallization did not occur in the first heating cycle, while in non-heat-treated specimens, it occurred at a temperature of 93–100 °C. Therefore, conducting the heat treatment of specimens at 100 °C was fully justified. Composite specimens showed a lower peak temperature of cold crystallization than the non-heat-treated matrix, and they were also characterized by lower activation energy. Fibers acted as a nucleating agent, accelerating the initiation of the crystallization process, but no significant differences in the obtained degree of crystallinity were observed between the composites and the non-reinforced materials (Table 5).

Specimens of polylactide that were not subjected to heat treatment showed in the first heating cycle a strong effect of enthalpy relaxation resulting from, among other factors, stress relaxation in the material. This effect practically disappeared in the second heating cycle (the effect of stress relaxation is an irreversible process). In contrast, specimens of polylactide subjected to heat treatment showed practically no relaxation effect in the first heating cycle. After cooling and reheating, the effect of relaxation, although more visible, was still very weak. Hence, the beneficial effect of heat treatment was the reduction of internal stresses and the concentration of stresses formed during the rapid cooling of injection-molded parts.

The glass transition temperature that was determined in the first heating cycle was in the range of 62 °C to 65 °C, while in the second cycle, it reached about 60 °C. However, no relationship was observed between the tendency of a change in the values of *T_g_* and the presence of fillers.

### 3.2. Experimental and Theoretical Density 

The true density of the tested composites was determined by measurements, and the theoretical density was calculated for the intended weight percentage converted into a volume fraction. Table 1 gives the fiber density values provided by the manufacturers that were used to calculate the theoretical density of the composites. The results of the measurements and calculations are given in Table 6. Comparison of the true (measured) density and theoretical (calculated) density of the tested composites indicates the occurrence of some discrepancies in the values obtained for the composite with a higher filler fraction. A lower value of the true density compared to the theoretical value may indicate a slightly lower fraction of fibers than intended. With the increasing volume fraction of fibers in the composite, it is increasingly difficult to ensure the precise dispensing of the filler into the matrix during the compounding process. On the other hand, a lower volume fraction of fibers relative to the intended one results in a weaker than expected reinforcing effect.

The simultaneous introduction of carbon and basalt fibers into the composite in equal weight fractions is a compromise between the specific properties (resulting from the density of materials) and the price of the fillers. The density of the obtained composites is expected to be on average 2% and 3.6% lower than the density of the composites with only basalt fibers or only glass fibers added in the amount of 15 wt % and 25 wt %, respectively. With the same weight fraction, the volume fraction of carbon fibers in the composite is slightly higher than that of basalt fibers.

### 3.3. Mechanical and Thermomechanical Properties

Another factor that significantly limits the technical application of amorphous PLA is its low glass transition temperature. As shown by the results of the DSC analysis (Table 4), this temperature is comprised in the range of approximately 60–65 °C, which means that an equally low heat resistance of this material is to be expected. In Table 7, the Vicat softening temperature was adopted as a measure of the heat resistance of the investigated composite matrices. This temperature could not be determined for the non-heat-treated composites, as they crystallized during the test, but it was determined for the unfilled and non-preheated matrix and for heat-treated materials.

**Figure 3:**
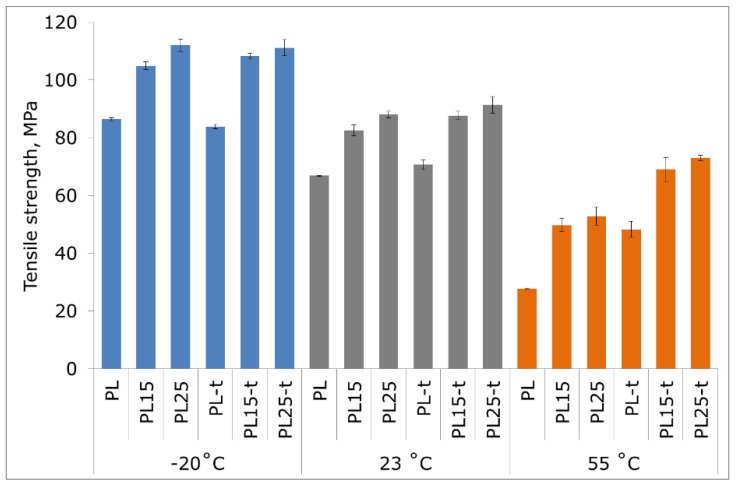
Tensile strength of tested materials at −20 °C, 23 °C, and 55 °C.

**Figure 4:**
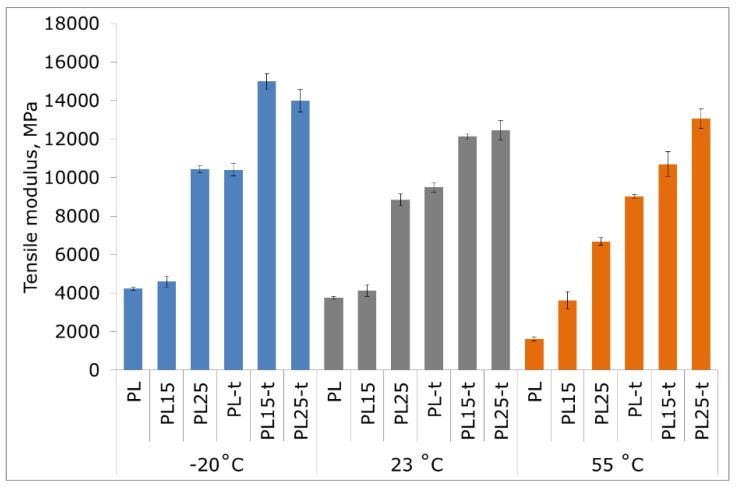
Tensile modulus of tested materials at −20 °C, 23 °C, and 55 °C.

The incorporation of fibers into an amorphous matrix had no special effect on the improvement of heat resistance. Only the presence of the crystalline phase ensured a physical blockage of deformations and the effect of synergic action with reinforcing fibers, reducing the possibility of movement of amorphous phase macromolecules, and thus contributing to a significant increase in heat resistance. This is also evident when the tensile strength of materials stretched at different temperatures is compared (Figure 3). The beneficial effect of the crystalline phase is mainly revealed at elevated temperatures. At 55 °C, the tensile strength and modulus of the heat-treated PL25-t is 51% and 45% higher, respectively, than an unfilled semi-crystalline matrix, and by as much as 165% and 710% higher, respectively, than an unfilled and non-heat treated PLA (Figure 3 and Figure 4). At both room temperature and low temperature, the elastic modulus of the tested materials with a high degree of crystallinity also assumes higher values compared to the values obtained in materials with a low degree of crystallinity. On the other hand, at the same temperatures (−20 °C or 23 °C), the values of the tensile strength of the materials before and after heat treatment are very similar to each other.

Comparing the obtained results with literature data for PLA composites with basalt fibers (15 wt %, 20 wt %, and 30 wt % fibers) [28] and glass fibers (15 wt % and 20 wt % fibers) [31], it can be concluded that the composites described in this paper are characterized by more favorable mechanical properties, and the tensile modulus of elasticity in particular. Also, in the work of Ying Z. et al., concerning amorphous and annealed PLA–basalt composites, the materials of similar and higher fiber loading show much lower tensile modulus and comparable tensile strength in comparison with the composites presented here [29]. It is worth noting that, while in the cited research works, silanes were used as proadhesive agents increasing the fiber–matrix adhesion, in the present research work, fibers were used without this adhesion-increasing agent. Hence, the further improvement of properties should be expected, as soon as a proper adhesive agent is selected and applied.

**Figure 5:**
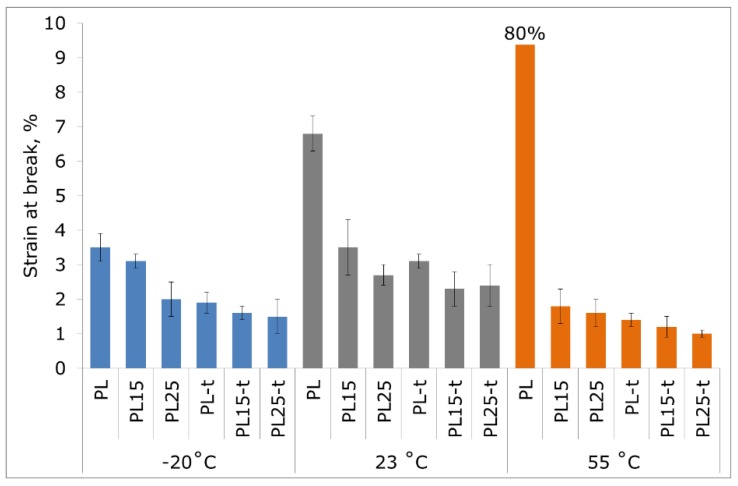
Strain at break of tested materials at −20 °C, 23 °C, and 55 °C. The value for non-heat-treated polylactide (PLA) at 55 °C reached 80%, which exceeds the plot range.

With the decrease in temperature, a typical phenomenon of the increase in strength and elastic modulus accompanied by a simultaneous decrease in the deformability of materials has occurred. The highest strain at break (Figure 5), regardless of the measurement temperature, was observed in unfilled and non-heat-treated PLA. 

The composites with a low fraction of the crystalline phase were also characterized by higher strain at break than the composites with a high fraction of this phase. The presence of the crystalline phase has contributed to a lower share of plastic deformation during stretching. This is also visible in the microscopic images of fractures formed in the tensile test.

### 3.4. Fractographic Investigation 

**Figure 6:**
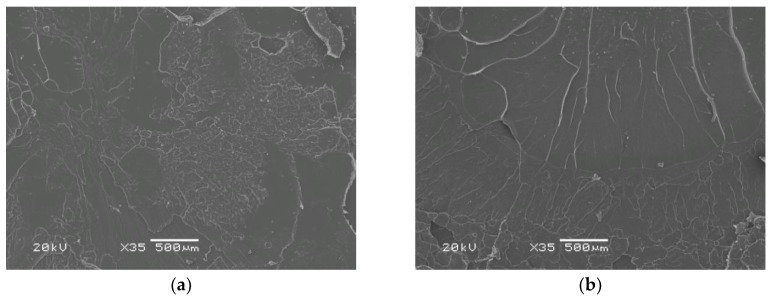
SEM images of tensile test fracture surfaces of polylactide specimens: (**a**) before thermal treatment (PLA); (**b**) after thermal treatment (PLA-t). Typical brittle fracture.

**Figure 7:**
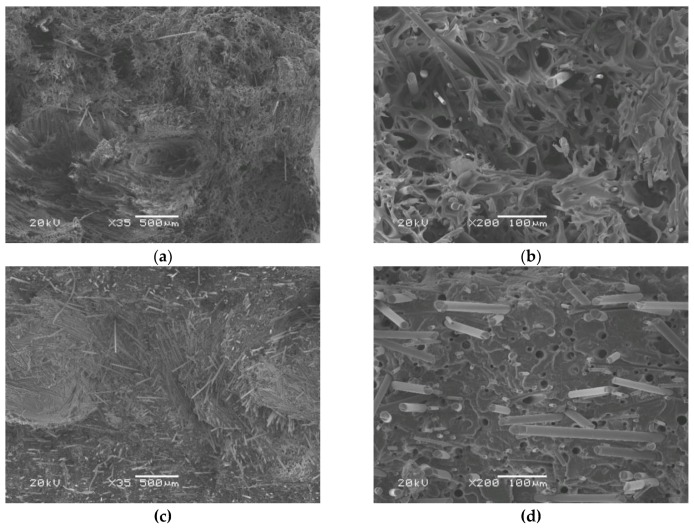
SEM images of tensile test fracture surfaces of hybrid composites with 25 wt % of the fibers: (**a**,**b**) before thermal treatment (PLA25) with visible signs of plastic deformation; (**c**,**d**) after thermal treatment (PLA25-t); brittle fracture.

Figure 6 and Figure 7 show SEM images of tensile fractures of non-heat-treated and heat-treated PLA and its composite containing 25 wt % of fibers. The fractures were obtained in a test carried out at room temperature. The non-reinforced PLA shows the typical symptoms of brittle fracture on the entire fracture area both before and after heat treatment. 

In contrast, the images of the composite clearly demonstrate that in the case of the heat-treated material with a high degree of crystallinity, the surface of the fracture is much less developed. In the images of the non-heat-treated composite, areas of a ductile nature are clearly visible (Figure 7a,b). This is due to a lower efficiency of fiber reinforcement in the material with a low fraction of the crystalline phase. 

In the presented SEM images, it is easy to notice the difference between large-diameter basalt fibers protruding more from the matrix and thinner carbon fibers. The effect of the fibers’ pull-out (long protruding fibers and relatively numerous rounded microvoids) is well visible, and fibers are not coated with a thin layer of the matrix. Therefore, this proves the need to strive for better fiber–matrix adhesion in the tested components.

### 3.5. Charpy Impact Resistance

**Figure 8:**
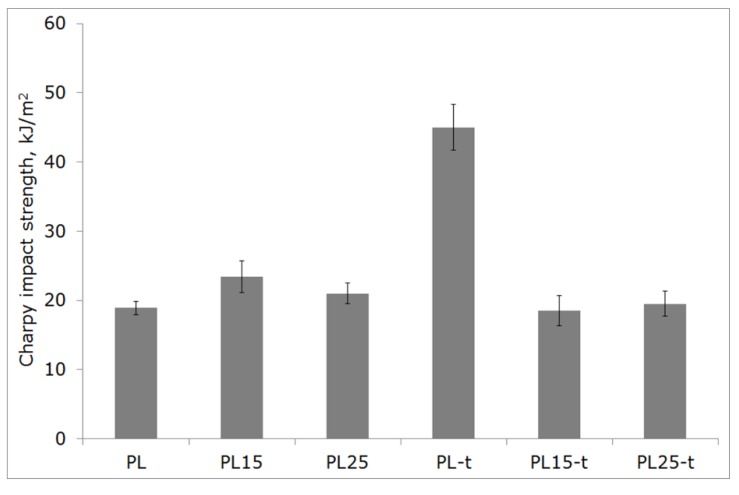
Unnotched Charpy impact strength of tested materials.

The brittleness of polylactide at room temperature also hinders its technical application as a material for components subjected to impact loads. However, in Figure 8, the beneficial effect of heat treatment on the impact strength of non-reinforced PLA is clearly visible. The impact strength has more than doubled. This effect may be due to the reduction of the stress concentration shown in the DSC tests or, in other words, to the relaxation process, which occurs during the annealing of moldings. 

The same beneficial effect was not observed in composites, where the impact strength of materials before and after heat treatment was similar, irrespective of a lower or higher fraction of fibers. The impact strength of all of the composites was at the same level as an impact strength of the non-heat-treated matrix. In this particular case, the agglomeration of fibers, which was visible in the fractographic images of composites (Figure 7c), might have a critical effect on the brittleness of the tested material.

### 3.6. Water Absorption

**Figure 9:**
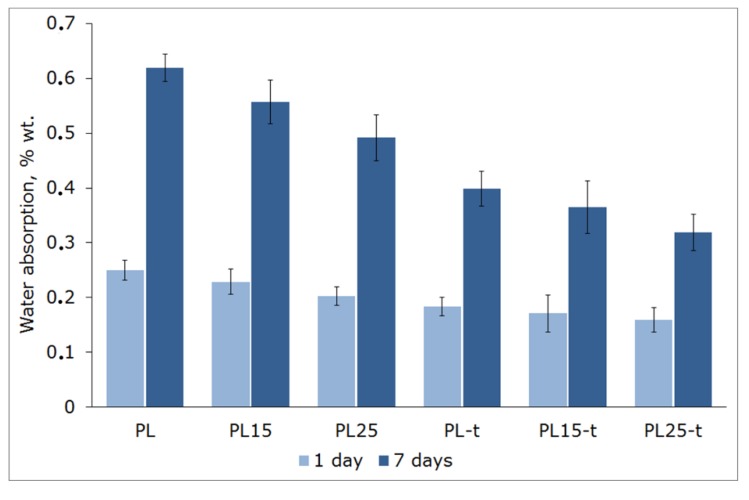
Water absorption of tested materials after one and seven days of soaking.

The drawback of amorphous polylactide is its water absorbability (Figure 9). It is worth pointing out that compared to polystyrene, which has similar mechanical parameters at room temperature, PLA is characterized by an obviously higher water absorbability after soaking for 24 h. After 24 h of incubation in water, polystyrene absorbs approximately 0.06% of water. However, the presence of a crystalline phase in heat-treated materials reduced this absorption rate. 

The absorption rate is also lower when non-absorbable fibers are introduced. They increase the dimensional stability of injection-molded parts and reduce fluctuations in strength parameters following the change in ambient humidity. 

It is worth pointing out that the decrease in water absorption that was caused solely by the increase in the PLA degree of crystallinity was more pronounced than the decrease obtained solely by the introduction of 25 wt % of the fibers. A combination of these two methods of water uptake reduction gave good results. It is more evident for longer incubation time. After seven days of soaking, the water absorption of the PLA25-t composite was 20% lower than for the neat heat-treated polylactide (PLA-t specimens) and as much as 50% lower than for the neat and non-heat-treated polylactide (PLA specimens). 

## 5. Conclusions

The presented research results indicate the great potential of hybrid polylactide composites as engineering materials. They may have properties superior to PLA composites with a high content of glass or basalt fibers (30–40 wt % of fibers of one type), especially when the values of elastic modulus and some specific density-related properties are compared. A complex of high properties was obtained especially in the case of composites with a total content of basalt and carbon fibers amounting to 25 wt %. The highest reinforcing effect was obtained in heat-treated composites. Heat treatment contributes to a significant increase in the degree of crystallinity of the tested materials, and thus to a significant increase in their heat resistance and elastic modulus, also at room temperature. A higher degree of crystallinity and the use of non-absorbable fibers reduce the water absorption rate, which is very important for the absorbable polylactide. Additionally, in heat-treated moldings, the beneficial effect of stress relaxation occurs in the areas of stress concentration. Moreover, polylactide, including also the polylactide reinforced with fibers that have only a limited nucleating effect, crystallizes slowly after injection into the mold. This adds to the cycle time. The use of additional heat treatment to enhance the crystallization process of the material is all the more purposeful in this case.

## Figures and Tables

**Table 1:** Properties of the fibers used to reinforce the composites (according to producer’s technical data).

Fiber Type	Density (g/cm^3^)	Tensile Modulus (MPa)	Tensile Strength (MPa)	Nominal Diameter (µm)
Basalt fiber (Basaltex, BCS17-6.4-KV16)	2.67	84	1880	7
Carbon fiber (Zoltek, PX35)	1.81	242	4137	17

**Table 2:** Composition of tested materials. PLA: polylactide.

Specimen ID	Matrix	Basalt Fiber Fraction (wt %)	Carbon Fiber Fraction (wt %)	Thermal Treatment
PL	PLA (Ingeo 2500HP, Nature Works)	-	-	NO
PL15		7.5	7.5	
PL25		12.5	12.5	
PL-t		-	-	YES (100 °C, 45 min)
PL15-t		7.5	7.5	
PL25-t		12.5	12.5	

**Table 3:** Test specimen injection molding temperature profile.

Specimen ID		Temperature (°C)					
	Feeder	Zone 1	Zone 2	Zone 3	Zone 4	Die	Mold
PL, PL15, PL25	45	190	200	210	210	210	20

**Table 4:** Characteristic temperatures at first heating–cooling cycle (I) and at second heating (II).

Specimen ID	*T_g_* (°C)		*T_cc_* (°C)		*T_m_* (°C)		*T_c_* (°C)
	I	II	I	II	I	II	I
PL	62	61	100	101	179	177	67/98
PL15	64	61	94	95	179	177	65/96
PL25	64	61	93	94	175/179	177	66/99
PL-t	64	61	-	101	179	177	66/104
PL15-t	65	61	-	95	178	177	65/100
PL25-t	64	60	-	94	178	177	66/96

**Table 5:** Degree of crystallinity determined at first heating–cooling cycle (I) and at second heating (II).

Specimen ID	*χ_c_* (%)	
	I	II
PL	18.1	20.0
PL15	19.6	21.3
PL25	19.1	27.1
PL-t	54.0	25.0
PL15-t	49.0	16.2
PL25-t	54.6	28.2

**Table 6:** Experimental end theoretical densities of the materials.

Method	Density (g/cm^3^)		
	PL	PL15	PL25
Experimental	1.220	1.302	1.341
Theoretical	-	1.305	1.368

**Table 7:** Heat resistance of the matrices and the composites after thermal treatment.

Specimen ID	Vicat Softening Point (°C)
PL	60
PL-t	162
PL15-t	166
PL25-t	168
